# The Animal Welfare Science of Working Dogs: Current Perspectives on Recent Advances and Future Directions

**DOI:** 10.3389/fvets.2021.666898

**Published:** 2021-10-15

**Authors:** Mia L. Cobb, Cynthia M. Otto, Aubrey H. Fine

**Affiliations:** ^1^Animal Welfare Science Centre, Faculty of Veterinary and Agricultural Science, The University of Melbourne, Melbourne, VIC, Australia; ^2^Penn Vet Working Dog Center, University of Pennsylvania School of Veterinary Medicine, Philadelphia, PA, United States; ^3^College of Education and Integrative Studies, California State Polytechnic University, Pomona, CA, United States

**Keywords:** animal welfare, dogs, human-animal interaction, science, sustainability, working dogs

## Abstract

Working dogs are prevalent throughout our societies, assisting people in diverse contexts, from explosives detection and livestock herding, to therapy partners. Our scientific exploration and understanding of animal welfare have grown dramatically over the last decade. As community attitudes toward the use of animals continue to change, applying this new knowledge of welfare to improve the everyday lives of working dogs will underpin the sustainability of working with dogs in these roles. The aim of this report was to consider the scientific studies of working dogs from the last decade (2011–2021) in relation to modern ethics, human interaction, and the five domains of animal welfare: nutrition, environment, behavioral interaction, physical health, and mental state. Using this framework, we were able to analyze the concept and contribution of working dog welfare science. Noting some key advances across the full working dog life cycle, we identify future directions and opportunities for interdisciplinary research to optimize dog welfare. Prioritizing animal welfare in research and practice will be critical to assure the ongoing relationship between dogs and people as co-workers.

## Introduction

Confidence in good animal welfare practices has been identified as critical to maintaining public support and the sustainability of industries dependent on animals (1, 2). Working dogs are prevalent around the world and fulfill many roles, adding social, cultural, and economic value to human lifestyles. They are valuable co-workers, providing labor that would be more costly for humans to do (3, 4), or performing specialized tasks that people are unable to accomplish, such as scent detection or as the focus of animal-assisted therapy (5, 6). Despite their value, many working dog providers only graduate around half of the dogs bred or recruited to their programs to operational working service, indicating inherent wastage (7).

Over the last 10 years, there has been growing scientific investment to better understand all aspects of working dog genetics, rearing, training, and functional performance in areas as diverse as scent detection, therapy, mobility, and safety with a view to improving canine performance, welfare, and program efficiencies [(7–11)]. Animal welfare science has also developed in the last decade, with the most recent update to the Five Domains Model adapted to include human-animal interactions, released in 2020 (12). Understood as *quality of life* or *how the animal is feeling*, animal welfare can be recognized as the lived experience of an animal. An animal's welfare is informed by positive or negative experiences across the domains of nutrition, environment, physical health, behavioral interactions; animal welfare scientists measure indicators of these experiences and the animal's mental state to assess animal welfare [(12–15)].

Hampton et al. (16) suggest that industries with strong scientific investment are more likely to retain community approval for their animal use, also referred to as social license to operate. The role of scientific research to inform modern animal management practices has also been identified as critical to industries reliant on animal use, including working dogs (7). Across private, government, assistance and service, racing, livestock herding and guarding working dog sectors, risk assessment may identify a generalized lack of transparency, stakeholder engagement and sharing of evidence-based best practices or standards to ensure the wellbeing of working dogs at the operational level (7). Where industry practices do not meet community expectations, the social license to operate may be revoked, resulting in industry disruption, or cessation or that type of animal use (16)). Examples from the last decade include interruption to greyhound racing and the phasing out of exotic animal circus performances in many locations globally (17, 18). Animal-reliant sectors that have transparency of animal care and management practices, demonstrate genuine engagement that leads to trust with their stakeholders (including the general public). Sectors which are science-informed appear more resilient to media exposés and loss of social license resulting in industry disruption (16). The ongoing use of working dogs is therefore more likely to be sustainable when operators have a strong record of independent scientific research and consequently function using evidence-based best practices that demonstrate how animal welfare is monitored transparently [(7), Hampton et al. (16)]. In the case of working dogs today, animal welfare largely reflects the interplay between three key components: the individual dogs, human attitudes and behaviors, and the physical environment, including facility management practices.

Recognition that dogs are sentient animals, possessing intrinsic value beyond their consideration as possessions, equipment or working contribution is being reflected in changes to legislation and politics globally (e.g., Australia, European Union, New Zealand, Canada, United States, and United Kingdom) (19–22). This shift is representative of a change in our relationship with these animals and the importance we place on their wellbeing and feelings (23). Although the scientific understanding of sentience and animal welfare science are interlinked concepts, the relative importance of species' characteristics is still being explored. For example, research to better understand cognitive abilities, evolution and selection, biological functioning, affective states, natural living, measurement of experiences, observation of behavior and social relationships, or other elements to reflect the lived experience of animals to inform animal management practices [(23–25)]. Concern has been expressed that animal welfare science has focused on optimizing performance and productivity of animals for the benefit of humans, rather than understanding the lived experience, needs and interests of animals [(26, 27)]. This may reflect the economic motivations tied to the sources of research funding [(27–29)]. For some industries, it could be perceived that scientific input is engaged with an exploitative motivation, rather than protective, with little focus to increase understanding, empathy and compassion toward animals [(25, 27, 30)].

Among the concerns in relation to the welfare of working dogs shared by media in recent years, the issues of animal consent and vulnerability appear to be gaining momentum. These issues have not only been observed in relation to working dogs in the last decade. For example, arguments have been made with regard to chimpanzees as vulnerable subjects in research on the basis of confinement, dependency and communication barriers [e.g., (31)]. This has extended into legal discussions, where animal protection by law has historically existed only to the degree that animal and human interests coincide (32). However, the last decade has given rise to cases where non-human animals have been identified as “sentient and vulnerable beings in need of a legal voice” and attributed rights, challenging law previously considered an anthropocentric institution [e.g., (33, 34)]. These trends across different disciplines reflect the attitude shift of concern for animals present among citizens. Identifying vulnerability leads to moral obligations and duties of justice (35). Industries reliant upon animals, including working dogs, will need to be pro-active and transparent in assuring their animal production and care practices do not disappoint community expectations if they wish to have sustainable participation of animals in these roles (1, 7, 36).

The contributions of research to working dog welfare over the past 10 years can be found in scientific publications across all the domains of animal welfare. Researchers engaged with, or based within working dog providers, are generating scientific evidence across fields as diverse as animal behavior, stress physiology, genetics, and technology to learn more about what working dogs need and want, and to optimize performance in the specialized tasks we require of them. Determining whether an animal use is acceptable is often complex, involving consideration of elements such as sociocultural, economic, environmental, both human and animal health, and other factors (37). Science provides a way to help us understand the mental and physical effects of animal use on the animal, informing practices, legislation and decisions relating to animal lives (15).

The aim of this report was to capture key scientific advances relating to the animal welfare science of working dogs discussed by the authors and colleagues at the Wallis Annenberg PetSpace Leadership Institute workshop in 2020. In this paper, we have identified and reviewed these scientific studies of working dogs from last decade (2011–2021), with a particular focus on their relation to modern ethics, human interaction and the five domains of animal welfare. Using this framework, we were able to consider the recent advances in understanding across the full working dog life cycle. This analysis has identified future directions and opportunities for interdisciplinary research to optimize the welfare and assure a sustainable co-worker relationship for people and working dogs.

## A Framework to Investigate Recent Working Dog Welfare Advances: Areas of Focus

### Modern Ethics

For decades, the major impetus in investigating and evaluating the roles of animals working with people has focused on the human portion of the equation. Although the past couple of decades have seen great progress in assessing the welfare of working dogs (7, 12), there continues to be a disparity in how these services are valued and evaluated from both the human and animal perspectives. It is evident that in the early years, even with good intentions, most of our expectations emphasized the value of these services for humans, overshadowing the impact of the work on the animals themselves (29). Today, we are seeing a stronger trend to assure reciprocal assessments on both sides of the service partnership. Animals that work with people should have the ability to form meaningful relationships in their lives and an ability to live their lives fully, irrespective of their work activities. In an optimal scenario, the animals' work activities should be enhancing their quality-of-life experiences.

It behooves those involved in training and providing services alongside working dogs to prioritize both ends of the leash to ensure welfare and the value of the experience is more reciprocal. Although some assume that working dogs enjoy their work, the animals are typically not asked if they want to participate in the work that they do. They are just engaged, with consent assumed. Although, it seems today that more attention is given to ascertain if the animal seems comfortable in their position, there continues to be a lag in objectively assessing the welfare of working dogs (29).

Numerous researchers and scholars of ethics and animal welfare have stressed many ethical concerns that professionals need to consider in working with service/assisted therapy animals [(38–43)]. It is important to appreciate that if these working experiences cause an animal to have little control over their daily life and bring discomfort, this can induce unhealthy stress. For example, Burrows (44) reports that, early on, some dogs that were used as service dogs for persons with autism were tethered next to the child for an unrealistic amount of time. Due to the lack of awareness by some of the families, Burrows (44) reported that the dogs within this study experienced undue stress from their interactions.

When addressing the welfare of working dogs, we must consider the ethical parameters of how to judge the process to make ethical decisions that are in the best interest of all those involved. A starting place is integration of a plan into the decision-making process so that we will act with a sense of integrity (45). Making appropriate decisions that consider the multi-dimensional aspects of these interactions for both humans and animals should be the cornerstone of initiating and guiding the process. Within the literature there are numerous ethical models that could be applied to one's decision making to effectively reflect on the work of service or therapy animals. Each of the models considers dilemmas from a distinct prism. The “ethics of care” approach strives to respect all parties involved by placing emphasis on sustaining relationships and the bond that is established (46). The primary focus of this model highlights the working relationship and the trust that is forged between the animal and all parties, as well as the animal's vulnerability. Within this model, whether the animal is provided with enriching quality of life experiences should be considered.

The “rights approach” primarily focuses on protecting and respecting the rights of all parties involved. This ethical approach assesses not only the human benefits derived from the relationship but also the pros and cons from the animal's perspective (47). Finally, the “utilitarian approach” uses a cost-benefit analysis that determines what we should act upon next, based on all the morally relevant consequences (usually harms and benefits for sentient individuals) of the actions available to us (48, 49). Within this model, it is simple to begin to address what are the costs that the animals might experience due to their work and daily experiences. The utilitarian approach does encourage evaluating the benefits that could also occur as a result of the actions. In following this approach, we must ensure that the costs and benefits are assessed objectively, for both the humans and working dogs alike. Such assessment should be robust, using multiple validated measures (physical, behavioral, and physiological) to ensure objective assessment of animal welfare (50).

The five domains (nutrition, environment, physical health, behavioral interactions, and their impact on the animal's mental state) of animal welfare (12) can provide a useful template to determine how the interactions and the working experience impact overall well-being. While not an ethical model, the “Five Domains Model” for animal welfare (12) offers an excellent perspective to assess the well-being of an animal by evaluating how the animal's physical and functional experiences impacts their emotional state. According to Peralta and Fine (46) the Five Domains model can be particularly useful in assessing the possible negative and the positive effects that the working relationship has on an animal's well-being (51). The model promotes the need to emphasize opportunities within each domain that lead to positive affective states (12). One needs to assess each of the domains to ascertain if any of the environmental, social, and physical interactions of working impact specific domains and directly or indirectly affect the animal's mental state.

These ethical decision-making frameworks can be applied to assess what should be considered to ensure that all parties' well-being is taken into consideration. As noted earlier, animals truly do not have a voice regarding their engagement. However, we believe that priority attention must be given to their welfare to assure quality of life. It is incumbent upon all practitioners who work with dogs, as well as researchers, to constantly ask questions about the human-animal relationships (established or being established) to ensure that the engagement is not one-sided, and that everyone's well-being is taken into consideration. This paradigm shift to recognize dogs as our co-workers and the application of ethical principles from human workplace settings (e.g., healthcare) to offer greater protection to working animals, reflects a change in moral understanding that has ethical implications for working with animals (52).

### Human Interaction

#### Attachment

While links between human attitudes, their relationship to behavior toward animals and the impact of human behavior on animal welfare has been studied in other animal-use contexts, such as farmed livestock (53, 54), there has been less focus on these relationships in relation to working dog and handler teams. However, beliefs and perception of people working alongside working dogs have been shown to be valuable in identifying animal welfare issues (55) and can be critical in shaping the success of some working dog partnerships, such as those between guide dogs and people who are blind or vision impaired (56).

Working dogs have been shown to perform differently for various handlers (57, 58), with implications for operational decision-making, such as working dogs having one or multiple handlers. This performance difference is likely underpinned by the interplay of canine and human personalities, as well as strength and style of attachment between the dog and handler (59, 60). Handler beliefs can impact canine work performance, as demonstrated by Lit et al. (61); when handler expectations were manipulated in an applied environment, alerts by scent detection dogs were impacted. Interestingly, another scent detection study that manipulated handler stress levels showed that working dogs showed improved performance when their handlers' anxiety levels were elevated (62). Such dog-human dyad studies often lack generalizability due to small sample sizes and are regularly taken from one workplace or population of dogs. An opportunity for future collaboration between multiple working dog providers, following the collaborative replication model established by programs such as Many Babies, Many Primates and newly established, Many Dogs (63, 64)), would allow for more robust testing of importance phenomena relating to the human-dog working team's performance and its relation to working dog welfare.

#### Training Methods and Equipment

Using only reward-based (positive reinforcement) training methods has been found to be more effective than use of aversive, compulsive, punishment-based (e.g., shock collars) or mixed methods. The use of only positive reinforcement results in more optimistic dogs with faster learning and more consistent behavioral responses who experience less pain and suffering, as well as reported lower incidence of aggression, problematic behaviors (e.g., unwanted barking), and symptoms of negative affect (65–69). Many people persist in using aversive methods when training their dogs, despite the known risks to canine welfare (66). A comprehensive review of modern working dog training has been provided by Hall et al. (70) within this special issue.

In many instances, the equipment used while working with dogs, such as collars, leads and harnesses, have not undergone much change in the past decade. The increasing use of pressure sensors, accelerometers and kinematics can offer new insights into how existing equipment impacts dogs when interacting with people [e.g., (71, 72)]. Given the emergence of new textiles and materials that may be stronger and lighter than traditional equipment, as well as nanotechnology, and smart textiles incorporating wearable electronics (73), we identify this as a future area for review and development.

#### Human Expertise

Humans are often flawed in accurately assessing our own skills and abilities. For example, the better-than-average-effect is a form of illusory bias exhibited across a wide range of competencies, such as driving, environmentalism, and even parenting (74). The effect is seen when people self-assess their capabilities upward, rating themselves better than reality and how others would rate them. Emerging evidence suggests a similar effect may be present in relation to how we perceive ourselves as providers of canine welfare (75). This highlights the importance for evidence-based education programs for all people involved with working dogs and role that external auditing should perform in quality control.

The Dunning–Kruger effect (76) is described in social psychology as the ignorance of ignorance. That is, people lack the knowledge and awareness to recognize what they don't know about a topic. The effect means that those with very little knowledge about something will often believe themselves to have high expertise in that area, preventing them from recognizing mistakes. Understanding how these aspects of social psychology relate to attitudes and behaviors toward dogs, as well-developing best practice, evidence-based professional knowledge transfer for people involved with working dogs is identified as an opportunity for future research.

### Nutrition

#### Provision of Food and Hydration

One of the basic tenets of animal welfare is the freedom from hunger and thirst (51, 77). The provision of adequate food and water is necessary for sustenance, but when considering the welfare of working dogs, this requirement must be viewed through a different lens. Rather than avoiding the negative welfare effects of inadequate nutrition, or settling for merely adequate nutrition (51), the focus should be on enhancing the positive impact of optimal nutrition. In addition, the knowledge base continues to expand and new information to support the working dog must be considered. Working dogs have increased nutritional demands due to the nature of their work. Detection and protection dogs often work in adverse environments and are engaged in physical activity that can lead to dehydration (78–80). Even 15 min of retrieving a ball can lead to fluid loss and detectable dehydration (81). The research over the last decade has particularly contributed to improving our understanding of optimizing hydration in working dogs.

Working dogs are selected for high motivation to engage in their trained task (e.g., searching for a trained odor or apprehending a fleeing suspect). When engaged in tasks that are rewarding or stimulating, these dogs will override the physiologic signals that drive thirst and are critical in preventing dehydration. Even mild to moderate dehydration impairs cognition, decreases alertness, and increases fatigue in humans (82). The effects of dehydration on cognition and fatigue have not been studied in the dog. Adequate hydration is also essential for control of body temperature. Unlike humans and horses, dogs do not regulate body temperature through sweating. Dogs rely on panting for heat exchange (83) and therefore can be at increased risk of heat-related injury when dehydrated (84). In working dogs, heat injury is recognized as a major and preventable cause of morbidity and mortality (85, 86). Hydration research represents an area of focus, due to the impact of inadequate hydration on performance and welfare and the ability to positively impact hydration in active working dogs.

The human partner of the working dog must be the advocate for the welfare of the dog, which translates to developing strategies to maintain and enhance hydration. One of the simplest approaches is to interrupt the dog during work to provide a hydration break. The dog may still be more focused on work than its physiology, therefore strategies to encourage drinking may be necessary. Although traditionally electrolyte replacement solutions were not recommended for dogs since they do not lose electrolytes through sweat (87), recent studies have suggested that electrolyte replacement solutions can be safe, palatable and may enhance heat tolerance in working dogs (79, 80). The benefit of electrolyte solutions does not appear to be a result of increased palatability and fluid consumption, because flavored water did not show the benefit and may lead to adverse effects (i.e., increased muscle damage) (80). The benefits of electrolyte solutions may be replacement of electrolytes lost in saliva during panting and in urine during exercise (79, 80, 88). On the other end of the scale, excessive water consumption can result in “water intoxication” and the associated dangerously low blood sodium and even death (89). Typically, physiological responses prevent continued water intake, but highly motivated dogs may override the signals, or may consume excessive water during swimming or playing with water (e.g., chasing a hose). This is another setting in which the welfare of the working dog will be directly impacted by the handler's awareness.

Like the requirement for hydration at a level commensurate with the work expected of a working dog, nutrition for optimal welfare extends beyond providing calories. In its simplest form nutrition should be a balance of protein, fat, carbohydrate, fiber, and essential vitamins and minerals to sustain life. For an active working dog, the physical demands alter the nutritional requirements (87). Protein requirements are increased to help build and support muscle that is being used in the work tasks (87, 90). For dogs that require endurance activities, higher fat content in the diet is required (87, 90). In addition to the type of food provided, the recommended frequency of feeding is based on the type of work. Dogs that compete in sprinting or intermediate distance activities may benefit from a 20 to 30% reduction in calories 24 h prior to activity. It is recommended that dogs undergoing vigorous activity are not fed in the 8 h prior to or immediately following the activity. Endurance athletes may require twice daily feeding (87). The understanding of working dog nutrition is a continually evolving field, and a comprehensive review is beyond the scope of this paper; for a recent review of working dog nutrition, see Zoran (90).

#### Quality of Food

Animal nutrition is a field prone to the application of current human dietary trends to animals. The motivation behind these feeding practices may be to appeal to human purchasers, but can also lie in an attempt to increase the nutritional benefits to the dog (91), conversely, the use of non-traditional diets may put both humans and animals at risk of disease. Two common feeding practices that have been associated with adverse health effects are the use of grain-free diets and raw meat diets. Although still controversial (92), studies suggest that some dogs fed a non-traditional diet (grain-free with non-traditional legume-based protein sources) have an increased risk of dilated cardiomyopathy (93, 94). Diets based on raw meat are popular among animal companion owners (91), however, the Center for Disease Control and Prevention (https://www.cdc.gov/healthypets/publications/pet-food-safety.html), the United States Food and Drug Administration (https://www.fda.gov/animal-veterinary/animal-health-literacy/get-facts-raw-pet-food-diets-can-be-dangerous-you-and-your-pet) and veterinary organizations (such as the American Animal Hospital Association [AAHA] https://www.aaha.org/about-aaha/aaha-position-statements/raw-protein-diet/ and the American Veterinary Medical Association [AVMA] https://www.avma.org/resources-tools/avma-policies/raw-or-undercooked-animal-source-protein-cat-and-dog-diets) have all issued statements warning against the use of raw pet foods due to the hazards of microbial contamination as well as challenges with creating an appropriately balanced diet. Diets for working dogs should be based on nutritionally sound formulations that are demonstrated to be safe for the dog and the canine handler/owner. One strategy to avoid unrecognized nutritional deficiencies and address the welfare benefit of providing a varied diet (12) may be to rotate diet formulations.

In addition to the basic nutrients, functional foods (those that provide benefits beyond nutritional value) and dietary supplements may have a role in supporting health and wellbeing of the working dog. Dietary supplements represent a rapidly growing industry and topic of great interest, with limited clinical trials. Most supplements are designed to reduce inflammation and improve joint health, a relevant impact for working dogs where osteoarthritis is a common occurrence (95). Currently, the supplements with the most scientific evidence of efficacy are the omega-3 fatty acids (96). The balance of omega-3 fatty acids is important for cognition and as an anti-inflammatory, particularly for management of osteoarthritis. Other functional foods may also have a role in supporting the wellbeing of working dogs (97) but more research is necessary. Beyond foods and dietary supplements, one of the most efficacious approaches to minimize inflammation and pain associated with osteoarthritis is weight control.

A greater problem in modern working dogs is not inadequate calories, rather, an excess of calories. Obesity is a frequent problem in pet dogs (98, 99) and is surprisingly common in working dogs. The optimal body condition score of pet dogs is between a 4 and 5 out of 9 (100). Working dogs' body condition score should be between a 3.5 and 4.5 out of 9. The impact of carrying excess weight is multi-fold. The added mass increases the effort and energy required for activity. Fat is an insulator that can reduce surface heat loss and increase the risk of heat injury. Additionally, adipose tissue is metabolically active and is responsible for the release of inflammatory cytokines that contribute to the progression of osteoarthritis and other inflammatory conditions. In a longitudinal study of Labrador retrievers, a difference in a body condition score of 5 out of 9 vs. 7 out of 9 translated to a lifespan of almost 2 years longer (101).

### Physical Health

The physical health of a working dog must be considered from the time of birth or recruitment, throughout the dog's working life and into retirement. Some breeds of dogs as well as individual dogs do not have the physical structure to safely participate in the required tasks of some working roles. For example, a brachycephalic dog that is unable to effectively pant will be at high risk for heat injury during exercise (102). Likewise, a dog with hip dysplasia will not have the structural stability to serve pain-free as a guide, mobility assistance, police, or search dog (103).

Preventive care is critical to maintain working dog health and an example of minimum requirements are described in the AAHA recommendations (104). The environment in which the dog works will dictate the specifics of care; however, all working dogs should have veterinary examinations at least annually. Disease prevention includes vaccination with the core vaccines as recommended by AAHA (105) and inclusion of vaccines for infectious diseases like leptospirosis, canine kennel cough complex and canine influenza based on individual, geographic and environmental risk factors (106). All working dogs should have a comprehensive parasite control program to address both internal and external parasites. Based on the mortality associated with gastric dilatation and volvulus documented in the US military working dog program (85), prophylactic gastropexy should be considered in large breed, deep chested working dogs. Current minimally invasive techniques (107), and limited complications (108) support the welfare recommendation to perform this elective procedure in dogs at risk. Other management decisions, such as spay or neutering working dogs, may also be associated with impacts to health and longevity in breeds such as Labrador and Golden Retrievers [e.g., (109–111)]. The role of the veterinarian in maintaining a low-stress environment during delivery of preventive care cannot be over emphasized. Despite the benefits of the medical care, aversive experiences associated with veterinary visits can negatively impact the welfare and subsequent performance of the working dog. Many of these dogs are highly arousable and minimal physical restraint or early implementation of chemical restraint or anxiolytics is now recognized as standard of care (112).

Physical fitness is an important welfare consideration (12). The implementation of a fitness program requires that the dog is physically capable of the exercises, the environment is safe for performance of the exercises and the training protocol creates a positive experience for the dog. A foundational fitness program has been described for working dogs (10). Any canine fitness program should include flexibility, body awareness, endurance (both cardiovascular and muscular), strength, and mobility. The intensity of the program should be gradually increased in response to objective assessments of the dog's performance, with safety for the dog and the handler paramount. A balanced fitness program will also include mental fitness as the dog learns new behaviors, develops resilience to environmental distractions and increases focus during the exercises (113). The benefits of fitness extend beyond the mental and physical stimulation associated with the training; a fitness program can aid in injury prevention, speed recovery from injury/illness and provide an opportunity for positive human-dog interactions.

### Environment

Working dogs can be deployed across a wide range of different environments, from therapy room, to snowy forest or hot desert. The welfare of the dog is dependent on the human partner, which translates to providing a safe location when not working (i.e., in a home environment, during transportation or kennel facility), recognizing early signs of overexertion, disease, dehydration and thermal stress. In the US, kennel facilities, whether in a home or in agency housing are required to meet accepted current USDA Animal Welfare Act guidelines. See Animal Code of Federal Regulations: Title 9, Volume 1 January 1, 2016 (https://ecfr.io/Title-9). Additional kenneling and care standards are under development (https://www.nist.gov/osac/dogs-sensors-subcommittee; http://www.asbstandardsboard.org/published-documents/dogs-and-sensors-published-documents/). In addition, environmental enrichment, access to exercise and play (with people and with other dogs) all enhance the welfare of the working dog (114).

#### Thermoregulation and Heat Injury

Heat injury can be localized, for example blistered paw pads from hot surfaces, or systemic hyperthermia from exertion, hot environments, or inability to effectively cool. Systemic hyperthermia can lead to various degrees of systemic insult from heat stress (discomfort and physiologic response) to heat exhaustion (mild to moderate dysfunction with dehydration and decreased cardiac output) to heat injury (elevated body temperature with organ injury) to heat stroke (115, 116). Traditionally, heat stroke is defined as an elevated body temperature (>40.6°C; 105°F) accompanied with signs of neurologic dysfunction and the risk of multiple organ dysfunction (115). During activity, working dogs have been reported to maintain and recover from body temperatures above 41.1°C (106°F) without evidence of heat injury (80, 117). Prevention of heat injury needs to focus on a safe temperature-controlled environment for the dog, control of heat generating activity, and effective heat exchange. A common breach in welfare occurs when a dog is left in a closed vehicle in a hot environment, whether inadvertently or through a failure of cooling systems (118). Some dogs are highly motivated by the mental stimulation of their work and this may override normal physiologic triggers that drive thirst (80) resulting in exertional heat stroke. The environmental temperature and humidity should be considered when planning dog training sessions or determining work cycles to decrease the risk of heat injury (119). Finally, diseases or dog training equipment that obstruct the flow of breath (e.g., laryngeal paralysis, tight muzzles), inadequate hydration and lack of physical conditioning will all predispose dogs to heat injury (117).

#### Transportation

Transportation is a common occurrence for many working dogs and has been shown to be stressful and resistant to habituation if familiarization does not occur *via* positive early exposure in life (120). Of particular importance to note is the regular occurrence of working dogs being forgotten and left in unattended vehicles for extended periods, leading to their death when heat and dehydration impact without sufficient ventilation, hydration or cooling in place. In response to the climate crises and global warming, vehicle transportation has been identified as a risk for dogs, even in areas not traditionally considered hot, such as the United Kingdom (121).

### Behavior

Behavioral issues are a major contributing factor to the high failure rates in working dog programs (8). Reducing behavioral wastage (the proportion of dogs bred or recruited to train that do not reach operational status due to their behavior) by improved assessment and tailored support for dogs will bring welfare benefits (122). Research considering the behavior of working dogs over the past decade has largely focused on tests to improve the selection and performance of working dogs, with the aim of increasing program success rates, currently reported to be ~50% across different working dog sectors (3, 7). This focus on behavior has included assessment of behavioral characteristics considered predictive of suitability to work (122, 123); the genetics of working dog behavior (124); maternal care in working dog breeding programs (125); and development and testing of cognitive skills [(126, 127)]. The use of technologies to capture and support behavioral observations such as activity monitoring and bio-metric sensors, in conjunction with algorithms (e.g., machine learning) to process large data sets are also being deployed with the goal of enhanced screening of working dogs (128).

Although some behavioral assessments report good predictive validity (42), aspects of research-driven behavioral assessment that may obfuscate their translation to industry practice include inter-rater reliability, and the reliability and construct validity of behavioral measures (129). Terminology used to describe behavior can also vary widely between and across industry sectors, potentially creating confusion for researchers, working dog trainers and handlers alike (7, 130). Some dogs that fail out of one program may be suitable for other careers, prompting programs to consider developing a co-operative approach (8). Not all dogs that fail to reach operational status are considered to exhibit behavior suitable for rehoming to non-working placements. Community attitudes and media attention have prompted changes in some sectors that historically euthanized or abandoned working dogs as an end point to their training or working life [e.g., Royal Australian Air Force Wilson: (131); US Military: Alger and Alger (132, 133)]. This indicates the influence of community attitudes and the media in driving industry change to retain social license to operate. However, without research reporting on the behavior and welfare of working dogs that have career-transitioned, it is unclear how well they adjust to rehoming away from training or work. This is an important future direction for investigation to extend our understanding of dogs bred or recruited to work to full-life cycle consideration.

Further work to identify and understand behavioral indicators of working dog welfare is needed. While many studies have sought to advance the “production of better dogs” (127), it is time to focus on extending our identification of behavioral indicators of affective state and welfare specific to working dog operational environments, kennel facility and home settings (134). The importance of drastic social and physical environment change inherent in many working dog programs has been identified as a welfare concern [e.g., (135)]. New findings in this area, particularly with consideration for the influence of dog personality and coping styles, would be useful to practitioners and regulators in guiding the development and implementation of best practice and standards. For example, identification of the behavioral cues of detection dogs that require rest breaks in airports or understanding how best to transition a young dog from puppy raising home to training kennel, would help guide regulations for optimal welfare during work. The roles of early socialization, provision of agency, and lifetime opportunities to play (with dogs and people) for the wellbeing of working dogs are also important area to investigate that are currently unexplored. Emerging technologies, such as those utilized in bioacoustics and precision livestock farming, may be useful tools in the remote monitoring of behavior and welfare in settings such as kennel facilities and private home environments [(136–138)].

### Mental State

Optimal rest and sleep are critical for working dogs. Sleep is associated with emotional state in sentient animals and is necessary for consolidation of learning, immune function, optimal performance and recovery to ensure longevity in working dog roles (139–142). Remote monitoring of canine sleep can be used to alert staff to disruption or change from normal sleep patterns that might impact animal welfare (143). For example, sleep deprivation has been shown to be detrimental to learning, decision making, and promoting negative affective states in rats and humans (144) and can also interfere with canine physiological stress responses such as cortisol (145). In addition to getting enough good quality sleep, it is critical for working dogs' social and mental needs to be met (51, 146).

The term *enrichment* has been widely used to describe animal care or management practices that help overcome deficits inherent in an animal's environment or social life. For example, Gfrerer et al. (147) report on the benefits of conspecific interaction for Swiss adult military dogs usually housed in isolation. Rather than interpreting this activity from the human perspective as a training or enrichment exercise, this compensatory social exposure might be reframed to reflect that its function is enabling the dogs to meet their psycho-physiological and behavioral needs for interaction with other dogs for mental wellbeing. It may be useful to reframe our thinking of social, environmental and mental enrichment as “meeting critical needs,” rather than perceiving such programs as non-essential extra, if resources allow.

The capacity for animals to engage freely with their environment under their own motivation is referred to as *agency* (148). Promoting agency in animals can improve behavioral diversity and have a positive effect on their welfare (149), but can prove challenging in some working dog settings. For example, it may not be appropriate for a working seeing-eye dog to explore dropped food or approach another dog to play. Nonetheless, identifying and supporting regular opportunities for working dogs to exercise agency and increase behavioral diversity in both environmental and social contexts is an opportunity for future studies. One activity that has been shown to induce positive judgement bias in dogs, is nosework (150). Letting dogs engage in olfactory-based sniffing activities resulted in them exercising autonomy and agency, resulting in increased optimism (150).

Working dogs have demonstrated long-term behavioral resilience after deployment in acutely stressful situations, such as the search and rescue dogs deployed at the site of the September 11, 2001 terrorist attacks (151). Other studies, such as that by Wojtaś et al. (152), suggest that rescue searches are stressful events to working dogs, demonstrated by elevation in salivary cortisol. The use of salivary cortisol in canine studies is widespread, yet it is a measure that can be influenced by a wide range of factors, making direct comparison between individual dogs and studies very hard (145). Our ability to differentiate dogs' acute and chronic cortisol responses as excitement or distress relies on interpretation offered by additional measures including behavioral observations and additional physiological indicators such as heart rate variability and immune function (135). This highlights the need for further investigation and assessment toward routine inclusion of multiple reliable and robust measures when assessing the welfare of working dogs.

## Discussion

### Full Life Cycle Consideration

Assessment of working dog welfare should occur routinely throughout working life (153–155), with regular reviews of exit data (when dogs are discontinued from training or retired from work) to look for patterns across time to identify other animal welfare concerns, relating to both physical and mental states [(156–158)]. These initiatives should include consideration for all environments and activities, including those outside of operational working sessions and with transparent surveillance and reporting across the full life cycle. This continuous improvement ethos should include adequate resourcing to be inclusive of breeding, rearing and/or recruitment; housing, transportation and husbandry practices; training techniques and dog training equipment; trainer and handler education; career change and retirement of working dogs.

### Scientific Research and Sustainability

Scientific information needs to be readily accessible to compete with other information reaching working dog industry stakeholders (29). Meaningful engagement and improved community outreach by researchers are needed to improve the uptake of research findings into evidence-based best practice. The knowledge deficit model of science communication traditionally used by scientists, centers on the assumption that ignorance is the basis for non-scientists in the community not adopting evidence-based best practice (159). Scientists following this model of science communication believe that one-way dissemination of their scientific knowledge to individuals and groups should be sufficient to prompt changes in their attitudes and behavior (160). The deficit model has been shown to be less effective than alternative bi-directional communication approaches that draw on the social sciences, such as participatory and community-based dialogue approaches (159, 161). This is particularly true in morally contentious areas such as the care and welfare of working dogs (29). When consulted, working dog industry workers have told the authors that scientists were not asking the questions they believed to be most important to industry (135). This is critical as research can be impacted by restricted access to working dog populations, and failure to win the trust of the industry through the way we communicate about our science may compound this.

Researcher access to working dog populations is limited and study cohorts are often statistically small (6). Analyses often draw on group averages, rather than group-based trajectory or latent class analyses widely used in human health research (e.g., Nagin et al. (162)). These techniques allow analysis of subgroups following similar response trajectories within a larger population, which might offer a more meaningful indication of individual preferences, responses and welfare [e.g., (163)]. There are limited opportunities for experimental manipulation with working dogs: kennel and animal management practices and training programs are generally well-established and successful dogs are required to meet operational and business requirements. This reluctance to change practices or participate in research, is seen in other areas where investment takes place over an extended time and the end product has high value (164, 165). Langston (164) notes that “*the role of industries in generating, shaping, and reinforcing norms, in addition to producing products, is often overlooked*.” In the non-profit sector particularly, where resources are limited, this results in experimental change being viewed as a risk to the success of the program.

The tendency for risk-averse industry groups to favor inaction highlights the need for more effective communication strategies between all working dog industry stakeholders if a sustainable outcome is to be achieved. A participatory, community-based research approach where industry representatives and researchers come together to formulate and answer questions of mutual interest is most likely to result in collaboration that fosters a shared purpose, improving uptake of research findings into evidence-based best practice (165, 166). Similar strategies in agricultural contexts found the participatory process gave farmers the analytical tools they needed to think critically and make informed decisions, improving their confidence when explaining the function of innovations to others and the desire to engage in sustainable change (165). This could be achieved by means of workshops to develop a schedule of research initiatives that are publicly, or government funded to better engage scientific researchers with the working dog industry to demonstrate the mutual benefits of collaboration.

Actively contributing to the development of this evidence base is possible by organizations and practitioners collaborating with scientific researchers. When this occurs, funders and researchers should insist that the animal experience is robustly assessed by multiple behavioral and validated physiological measures (29). This change would serve to help balance our understanding of the animal experience in working settings, where historically we have emphasized the human outcomes. Greater interdisciplinary collaboration between researchers (i.e., including animal welfare scientists in working dog research design and teams) will enable this, and result in greater uptake of research findings into practice. Ideally, all granting bodies who fund exploration of the possible benefits to people from working dogs should also fund and require that the working dogs' physical and mental experiences be reliably and robustly monitored and reported.

## Conclusion

Assuring all stakeholders, including the general community, that the welfare of working dogs is positive will be fundamental to retaining social license to operate across the varied working dog sectors. Good, transparent animal management practices informed by independent science to assure positive animal wellbeing will be needed to underpin a sustainable partnership working with animals in this way. Our understanding of animal welfare science and working dog performance have grown rapidly in the last decade. However, many aspects of working dog welfare have not been studied robustly and are ripe for research, innovation, and improvement.

Opportunities to make valuable contributions to improving the welfare of working dogs through further research have been identified across five domains of animal welfare in this analysis. Scientists working in this field can collaborate, within and between disciplines, to improve the validity of their work by studying composite dog populations and exchanging experiences between working dog sectors. In addition, researchers who to familiarize themselves with updated science communication strategies will have greater success in seeing their work translate to improved industry practices. Psychological studies show us that people tend to assume we are better at things than we actually are. Knowing this, there exists a responsibility to assure the positive welfare of working dogs. This can be achieved by committing resources to study the welfare of working dogs, the use of external auditing, and good science communication that enables practitioners to help shape and stay up to date with new working dog welfare science research.

Regular evaluation and adjustment of practices is essential so that the evidence gained through animal welfare science research can guide best practice and standards. Providing working dogs with positive life experiences (good physical and mental animal welfare) is likely to share the positive consequences observed when farm animal welfare is improved (167). This includes better performance, program efficiency, staff satisfaction, social, and economic benefits. Most importantly, it provides the animals involved with a good life that is worth living. This will be essential for people and dogs to sustain a co-worker relationship that retains social license to operate and respects animal vulnerability in a manner that is not detrimental to the welfare of working dogs.

## Author Contributions

MC, CO, and AF contributed to this manuscript's concept and provided content, edits, and review. All authors approved the final manuscript.

## Funding

As part of the Wallis Annenberg PetSpace Leadership Institute Fellowship initiative, funding for the publication of this article was provided by Wallis Annenberg PetSpace.

## Conflict of Interest

The authors declare that the research was conducted in the absence of any commercial or financial relationships that could be construed as a potential conflict of interest.

## Publisher's Note

All claims expressed in this article are solely those of the authors and do not necessarily represent those of their affiliated organizations, or those of the publisher, the editors and the reviewers. Any product that may be evaluated in this article, or claim that may be made by its manufacturer, is not guaranteed or endorsed by the publisher.
